# Investigation on the Enhanced Oxidation of Ferritic/Martensitic Steel P92 in Pure Steam

**DOI:** 10.3390/ma7042772

**Published:** 2014-04-03

**Authors:** Juntao Yuan, Ximao Wu, Wen Wang, Shenglong Zhu, Fuhui Wang

**Affiliations:** 1 State Key Laboratory for Corrosion and Protection, Institute of Metal Research, Chinese Academy of Sciences, Shenyang 110016, Liaoning, China; E-Mails: jtyuan@alum.imr.ac.cn (J.Y.); slzhu@imr.ac.cn (S.Z.); fhwang@imr.ac.cn (F.W.); 2 Tubular Goods Research Institute of China National Petroleum Corporation, Xi’an 710077, Shaanxi, China; 3 State Grid Liaoning Electric Power Research Institute, Shenyang 110006, Liaoning, China; E-Mail: wuximao69@163.com

**Keywords:** Fe–Cr alloys, high temperature oxidation, steam oxidation, TGA

## Abstract

Oxidation of ferritic/martensitic steel P92 was investigated in pure oxygen and in pure steam at 600–800 °C by thermogravimetric analysis (TGA), optical microscopy (OM), scanning electron microscopy (SEM), and X-ray diffraction (XRD). The results showed that the oxidation of P92 was significantly enhanced and multilayer scale with an outer iron oxides layer formed in pure steam. At 700 °C, the gas switch markedly influenced the scaling kinetics and scale microstructure. It was supposed that the higher affinity of iron to steam would be attributed to the enhanced oxidation of P92 in pure steam, and the much easier transport of hydroxyl would account for the significant difference induced by gas switch.

## 1. Introduction

In recent years, the effect of water vapor on the oxidation of Fe–Cr alloys has been studied extensively. It is indicated that addition of water vapor to oxidizing gas would significantly enhance the oxidation rate of Fe–Cr alloys with mediate Cr content compared to that in dry gas and result in rather different scale structure [1]. The primary reason is that water vapor causes the transformation of protective Cr-rich oxide scales into non-protective Fe-rich oxide scales, termed as “breakaway oxidation” [2–4]. Chromium evaporation in terms of CrO_2_(OH)_2_ has been preferably applied to interpret the breakaway of protective chromia scale in water vapor containing environments, especially in wet oxygen [5–7]. However, chromium evaporation in low-oxygen-level environments (e.g., pure steam) would be neglected since the formation of volatile CrO_2_(OH)_2_ needs the participation of oxygen [8,9]. In pure steam environment, breakaway oxidation was also observed in our previous work [10]. In this perspective, the breakaway of protective chromia scale might be attributed to the local failure like cracking [4].

Oxidation of 10%Cr steels showed a slightly different behavior. After oxidation in dry gases, a thin protective scale was formed. In wet gases, multilayer oxides consisting of an outward-growing iron oxides layer and inward-growing Fe–Cr oxides layer always form even after short-term exposure [11]. Breakaway oxidation phenomenon has not been observed in the scaling kinetics. This suggests a rather different initial oxidation mechanism from the breakaway oxidation; however, it is still open to debate.

Automatic recording balance make it possible to get a continuous record of the reaction kinetics and, in this way, many details come to light that are hidden by other methods [12]. The present work obtained the short-term continuous oxidation kinetics of ferritic/martensitic steel P92 by introducing pure steam into Thermogravimetric Analysis (TGA) system, and investigated the microstructure and chemical compositions of oxide scales by Optical Microscopy (OM), Scanning Electron Microscopy (SEM), Energy Dispersive Spectrometer (EDS), and X-ray Diffraction (XRD). The effect of gas switch on the oxidation behavior was also investigated by TGA to reveal the influence of water steam on the scale growth rate of P92 *in situ*. Based on the results, the mechanism of steam oxidation was discussed.

## 2. Results

### 2.1. Oxidation in Pure Oxygen

**Figure 1.**
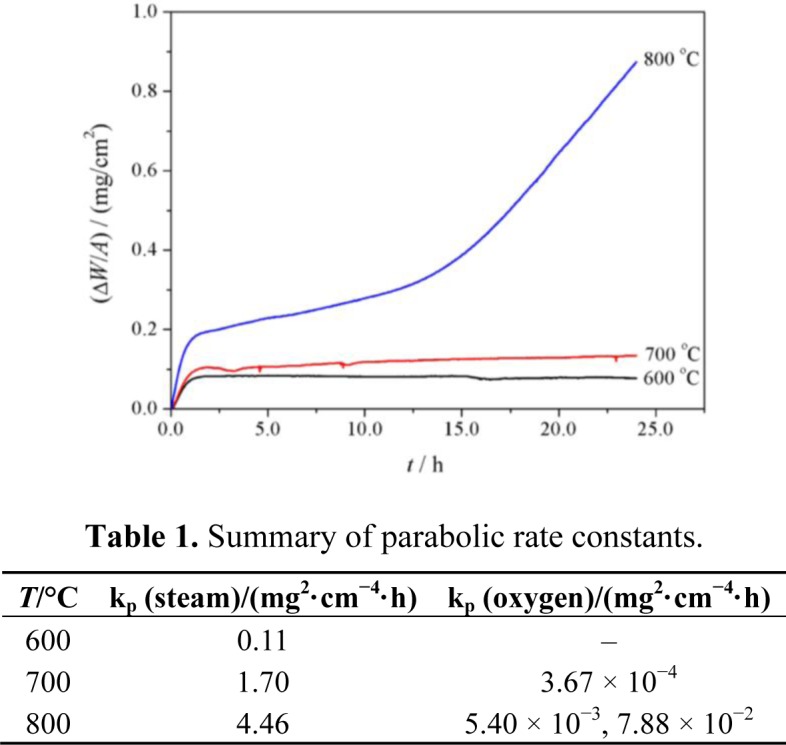
Oxidation kinetics of P92 steel in pure oxygen.

Figure 1 shows the oxidation kinetics of P92 exposed to pure oxygen at 600–800 °C. It is evident that oxidation kinetics is temperature-dependent. Mass gains are significantly increasing with temperature elevated. At 600 and 700 °C, the mass gains are extremely small and the oxidation kinetics follows the near-parabolic rate regime. At 800 °C, breakaway kinetics can be seen after the initial relatively slow scaling stage. Oxidation kinetics were modeled using parabolic rate law as described as (ΔW/A)^2^ = k_p_·*t*, where (ΔW/A) is the mass gain (mg/cm^2^), k_p_ is the parabolic rate constant (mg^2^/cm^4^/h) and *t* is the exposure time (h). All the parabolic rate constants are summarized in Table 1.

**Figure 2.**
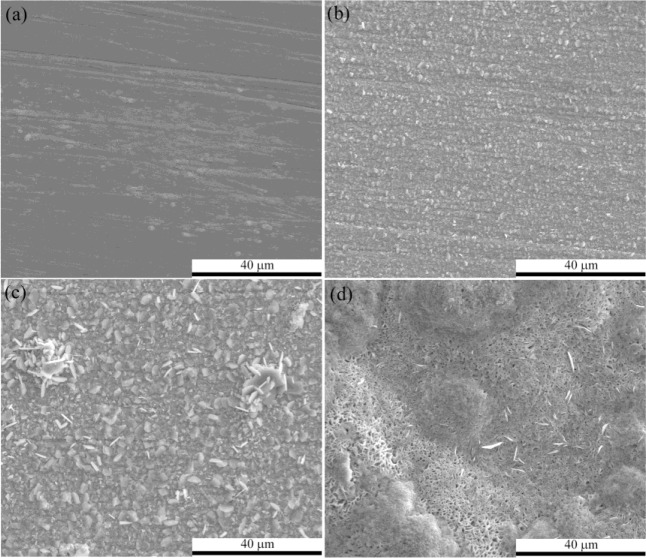
Surface morphologies of the flat scales formed on P92 after 24 h oxidation in pure oxygen at (**a**) 600 °C; (**b**) 700 °C; and (**c**) 800 °C; (**d**) Top-view image of surface nodules formed in pure oxygen at 800 °C.

Surface morphology of P92 after 24 h oxidation in pure oxygen was dependent on temperature as revealed in oxidation kinetics. At low temperatures (600 and 700 °C), relatively flat oxide scale with visible grinding marks and few Fe-rich nodules can be seen. As temperature rose up to 800 °C, large Fe-rich nodules became predominant, which was in accordance with the much greater scaling rate as suggested in oxidation kinetics. Figure 2a–c presents the morphologies of flat scales formed at studied temperatures. There is evidence that the flat scale thickened (because the grinding marks in the flat scale region disappeared gradually) as temperature rose and oxide ridges can be clearly seen at 800 °C. The morphology of oxide nodules formed at 800 °C is shown in Figure 2d, and indicates that it is covered by porous iron oxides.

**Figure 3.**
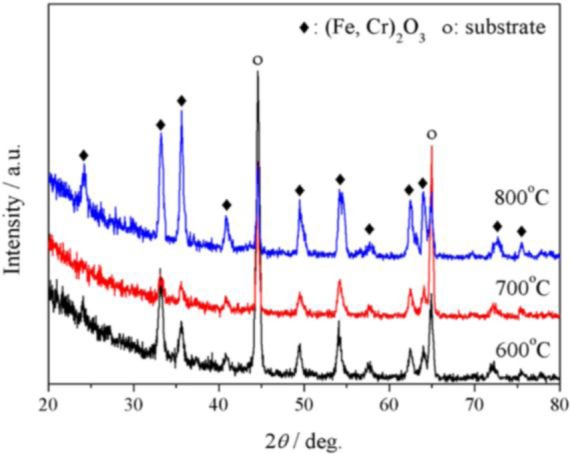
Grazing Incidence XRD (GIXRD) patterns of surface oxide scales formed on P92 after 24 h oxidation in pure oxygen.

Figure 3 displays the GIXRD patterns of P92 after 24 h oxidation in pure oxygen. At all temperatures, the oxide scale was composed of corundum-type oxides, noted as (Fe,Cr)_2_O_3_. The diffraction peaks were close to standard peaks of Cr_2_O_3_ at low temperatures, while they were near to standard peaks of α-Fe_2_O_3_ at 800 °C. At 800 °C, little amount of spinel oxides were also detected.

**Figure 4.**
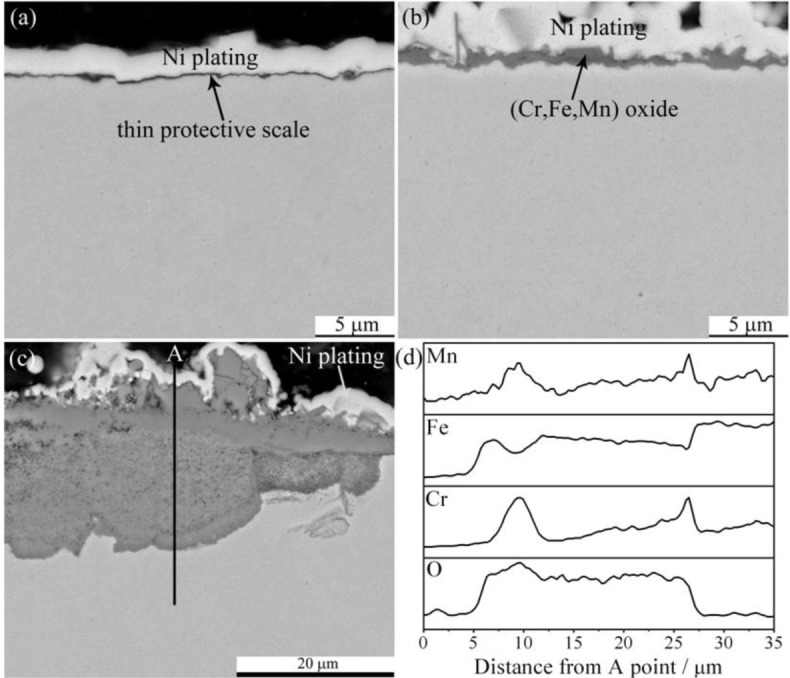
Cross section morphologies of base oxide scales formed on P92 after 24 h oxidation in pure oxygen at (**a**) 600 °C; and (**b**) 800 °C; (**c**) Cross section image of oxide nodules formed at 800 °C; and (**d**) chemical composition distribution along the line indicated in (**c**).

The cross section morphologies also showed the temperature dependence. Figure 4 presents the cross section morphologies at 600 and 800 °C, element distribution profile is also given there. The oxide scale grown at 600 °C (Figure 4a) was extremely thin, and few small iron-containing nodules can be seen. There was evidence that the oxide scale formed at 800 °C was embedded with large protruding nodules and concave craters. The base scale grown at 800 °C is shown in Figure 4b, which was thicker in some extent compared to that at 600 °C and mainly consisted of (Cr,Mn) oxides. The large oxide nodules formed at 800 °C is present in Figure 4c. The EDS result (Figure 4d) indicates that the protruding nodules were iron oxides while the concave craters were composed of (Fe,Cr,Mn) oxides. It is interesting that there were thin Cr-rich layers at nodule/crater and crater/substrate interfaces. It would be reasonable to consider the protruding nodules as Fe_2_O_3_, the thin base scale as Cr_2_O_3_, and the concave craters as (Fe,Cr,Mn)_3_O_4_.

### 2.2. Oxidation in Pure Steam

**Figure 5.**
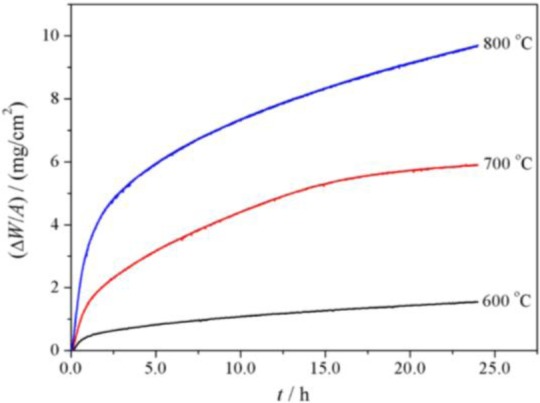
Oxidation kinetics of P92 steel in pure steam.

Figure 5 shows the oxidation kinetics of P92 in pure steam. It is clear that the mass gains in pure steam at a given temperature were approximately 20–40 times than those in oxygen. As temperature increased, the oxidation rate was significantly enhanced. In order to examine the kinetics regime, the time exponent of power function described as (ΔW/A) = k·*t^n^* were determined by plotting ln(ΔW/A)~ln*t*. The time exponents were 0.42, 0.49 and 0.38 for 600–800 °C respectively, suggesting that the scaling rate regime was near-parabolic. The parabolic rate constants are also given in Table 1. It is indicated that the oxidation rate of P92 in pure steam at a given temperature was several orders of magnitude greater than that in oxygen. In pure steam, the temperature-dependence of parabolic rate constants were modeled using Arrhenius equation, and the apparent activation energy was determined as 147.9 kJ/mol. This value was consistent to the oxidation activation energy of T91 in Ar + 10%H_2_O at 600–700 °C [13].

**Figure 6.**
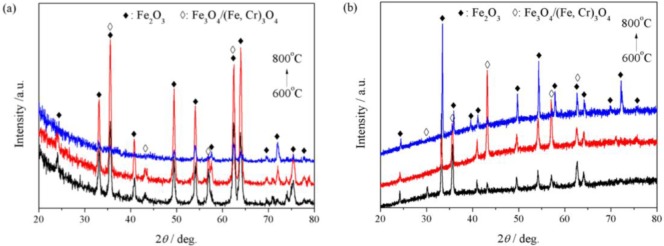
(**a**) GIXRD (incidence angle = 0.5°) patterns of the outermost surface oxides; and (**b**) XRD patterns of the outer surface oxides formed on P92 after 24 h oxidation in pure steam.

The outermost oxide scales formed on P92 after 24 h oxidation in pure steam were identified by GIXRD technology. Figure 6a presents the GIXRD patterns. It is suggested that the outer scales grown at 600 and 700 °C were composed of Fe_2_O_3_ and spinel oxides possibly as Fe_3_O_4_ and/or (Fe,Cr)_3_O_4_. At 800 °C, the exclusive diffraction peaks of spinel oxides at the position 2θ = 30.1°, 43.1° and 57.1° disappeared, indicating the absence of spinel oxides in the outer scale. Figure 6b shows the XRD patterns detected using X-ray with upright incidence angle. It can be seen that the diffraction patterns were very similar to GIXRD patterns, except that little amount of spinel oxides were detected at 800 °C.

**Figure 7.**
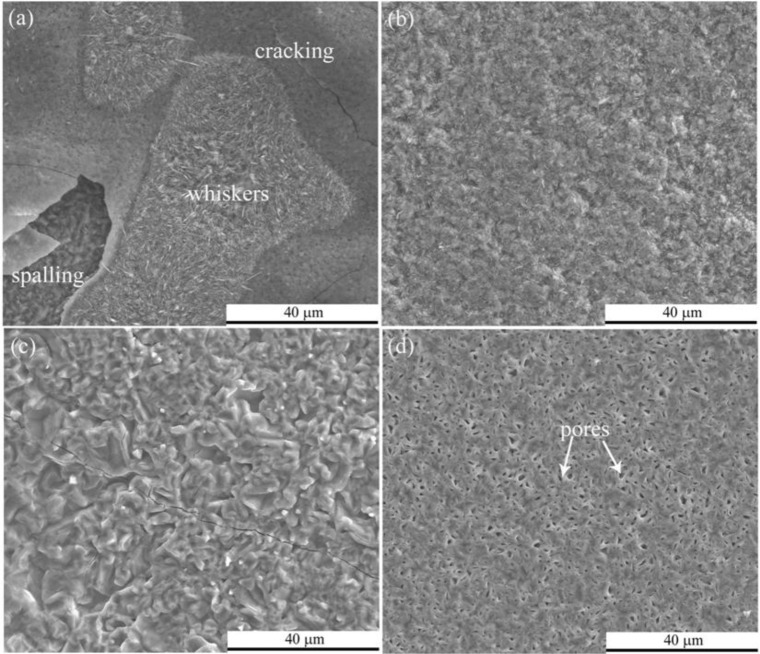
Surface morphologies of oxide scales formed on P92 after 24 h oxidation in pure steam at (**a**) 600 °C; (**b**) 700 °C; and (**c**) 800 °C; (**d**) showing the morphology of sub-scale after exfoliation of outer oxides at 600 °C.

Investigating the surface morphologies after 24 h exposure in pure steam by SEM, we found several characteristics. First, considerable exfoliation was observed at 600 and 700 °C, while only cracks were observed at 800 °C. Second, surface morphologies of outer scales varied with exposure temperature. As temperature increased, oxide whiskers were suppressed, as indicated in Figure 7a–c. A plenty of pores can be seen in the top-view images after the exfoliation of outer scale, as shown in Figure 7d. As revealed by GIXRD patterns, the outermost scale could be considered as Fe_2_O_3_, the sub-scale as Fe_3_O_4_.

**Figure 8.**
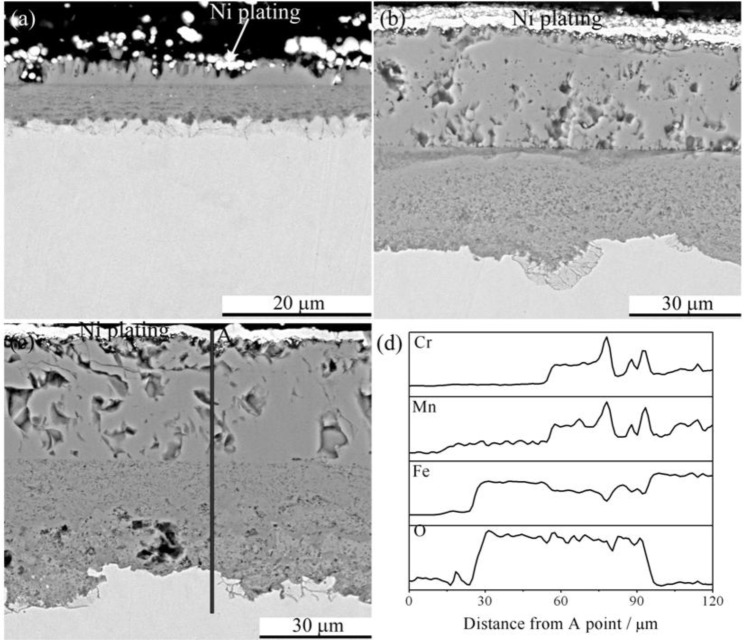
Cross section morphologies of base oxide scales formed on P92 after 24 h oxidation in pure steam at (**a**) 600 °C; (**b**) 700 °C; and (**c**) 800 °C; and (**d**) chemical composition distribution along the line indicated in (**c**).

Cross section morphologies of P92 after 24 h exposure in pure steam are shown in Figure 8a–c. At all temperatures, double-layer oxide scales were observed. The outer layer was somewhat thicker than the inner layer at 600 °C, while the thickness of these two layers tended to be equal as temperature rose up to 800 °C. Element distribution profile by EDS indicated that the outer layer was iron oxides while the inner scale was (Fe,Cr,Mn) oxides in all cases. Figure 8d shows the EDS line scan through the scale formed at 800 °C. It is noted that there was enrichment of Cr in the region near the interface between the inner layer and substrate.

### 2.3. Oxidation by Switching Gas at Interval

**Figure 9.**
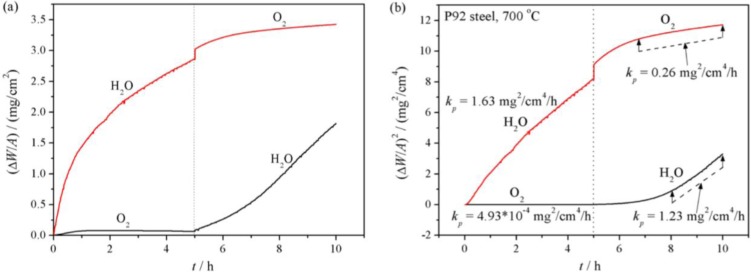
(**a**) Oxidation kinetics and (**b**) parabolic fitting of P92 in two-stage exposures at 700 °C.

Figure 9a shows mass gain data of P92 during isothermal oxidation at 700 °C with *in situ* gas switching (from pure steam to pure oxygen, or contrarily) every 5 h without intermediate cooling. In the “oxygen then steam” case, once oxygen was switched by steam, oxidation kinetics suddenly increased to a great extent. While in the “steam then oxygen” case, the oxidation rate was significantly decelerated when steam was switched by oxygen. Modeling the oxidation kinetics by parabolic law, the parabolic rate constants were taken from the parabolic plot as shown in Figure 9b. During the first oxidation stage, the bulk P92 showed similar parabolic rate constants as described in Table 1. But after gas switching, the rate constants were rather different. For the oxidation in pure steam, the rate constant in the second stage was slightly lower than that in the first stage. While for the oxidation in pure oxygen, the rate constant in the second stage was rather greater than that in the first stage.

**Figure 10.**
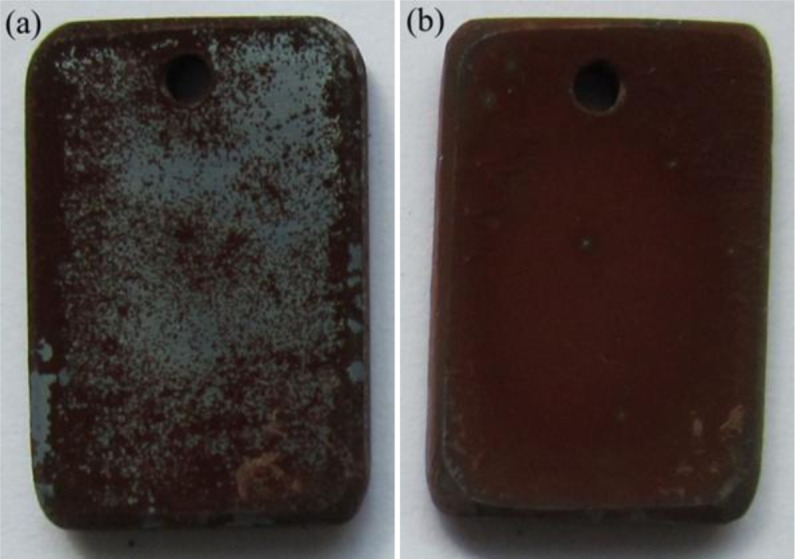
Macroscopical appearance of oxidized P92 specimens after two-stage exposure at 700 °C: (**a**) O_2_ then H_2_O; and (**b**) H_2_O then O_2_.

After two-stage oxidation, the appearances of oxidized specimens in two cases were rather different. Dark red and gray oxides can be seen on the specimen oxidized in the “O_2_ then H_2_O” case (Figure 10a), while the specimen oxidized in the “H_2_O then O_2_” case was wholly covered by dark red oxides (Figure 10b).

**Figure 11.**
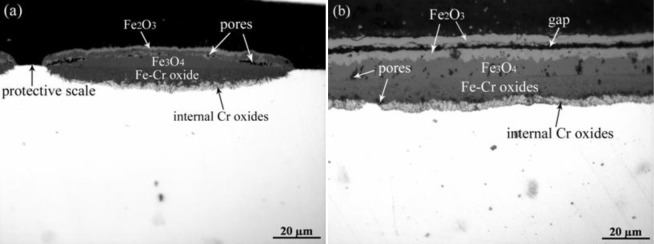
Optical Microscopy (OM) cross section images of P92 after two-stage exposure at 700 °C: (**a**) O_2_ then H_2_O; and (**b**) H_2_O then O_2_.

According to the oxidation kinetics, it can be speculated that the scale grown in the first-stage exposure in oxygen was mainly composed of thin protective (Fe,Cr)_2_O_3_ layer, the subsequent exposure in pure steam significantly enhanced the oxidation by prompting the formation of non-protective Fe-rich oxides. The OM images of the specimen after exposure in the “O_2_ then H_2_O” case is present in Figure 11a, it is evident that large oxide nodules with three-layer structure were embedded in the thin protective scale. At the edge of the specimen, thin protective scale disappeared. EDS line scan through the large oxide nodule indicated that the outer protruding layer was iron oxides, while the crater layer was (Fe,Cr,Mn) oxides. It is reasonable to infer that the three layers are hematite, magnetite, and (Fe,Cr,Mn) oxides form the oxide/gas interface to the oxide/steel interface. In addition, some amount of internal Cr oxides with dark contrast exists at the oxide/steel interface.

After oxidation in pure steam, double-layer scale with an outer Fe oxide layer and an inner (Fe,Cr,Mn) oxide layer was found, similar to the observation mentioned in Section 2.2. The subsequent exposure in oxygen reduced the oxidation rate to a great extent and led to a four-layer scale structure as shown in Figure 11b. Combined with EDS analysis, the outer layers with light gray contrast could be considered as hematite, the sub-scale with relatively dark gray contrast as magnetite, and the inner layer with the darkest contrast as (Fe,Cr,Mn) oxides. The hematite layer is separated into two layers by a continuous gap. It is worth noting that the thickness of hematite layer is slightly greater than the magnetite layer.

## 3. Discussion

Based on the experimental results, it could be found that oxidation behavior of P92 was significantly dependent on the oxidizing gas and temperature in short-term exposure. It also indicated that pure steam increased the critical chromium content required to form continuous external chromia scale, consequently prompted the formation of iron oxides and internal Cr oxides.

For the oxidation Fe–Cr alloys, the affinity of metal element to oxygen determines the stability of its oxide. According to ΔG°-*T* diagram [12], Cr_2_O_3_ is much more stable than iron oxides including Fe_2_O_3_, Fe_3_O_4_ and FeO. For the oxidation of metal, the thermodynamic stability of its oxides would reveal the possibility of reaction, but would not determine the reaction kinetics. During the initial oxidation of Fe–Cr alloys, Fe and Cr on the specimen surface would be oxidized simultaneously, so that the oxide scale would be determined by the relative oxidation rate of Fe and Cr. Here, the oxidation rate contains the concentration of reactant and transport parameters. For a Fe–Cr alloy with given chromium content, once the relative oxidation rate of Cr is faster, Cr would be oxidized selectively.

Oxidation of pure metals considered as extreme conditions will be discussed. In order to avoid the influence of carrier gas on the oxidation process, only the cases whereby inert gases were used as carrier gases are considered. Reviewing the published literatures, it can be found that the presence of water vapor remarkably accelerates the oxidation of iron [14,15], reduces the oxidation of nickel [16,17], and slightly increases [18] or does not affect the oxidation of chromium [19]. From this perspective, it can be speculated that water vapor would lower the relative oxidation rate of Cr in Fe–Cr alloys while improve the relative oxidation rate of Cr in Ni–Cr alloys. In consequence, the selective oxidation of chromium in water vapor would be suppressed in Fe–Cr alloys while prompted in Ni–Cr alloys. In other words, the critical chromium content in Fe–Cr alloys required to form protective chromia scale would increase in water vapor containing gases, as observed in many works [17,20,21]. Essuman *et al*. [22] also considered that water vapor enhanced the internal oxidation of Cr in Fe–Cr alloys, possibly due to the result of water vapor affecting the solubility and/or the diffusivity of oxygen in the alloy.

In the two-stage cases, the oxidation behavior of P92 was significantly influenced by the gas switch. After a slower scale-growing process in oxygen, the introduction of steam sharply increased the scaling rate and markedly transformed the protective scale formed in oxygen to non-protective iron-rich oxides. The breakaway of protective scale might be induced by the hydrogen effects generated in the oxidation process in steam [4]. Once the chromium concentration at the scale/steel interface cannot heal the breakdown of protective scale, the outward transport of iron ions and inward diffusing of hydroxyl ions would be prompted. In the “H_2_O then O_2_” case, multilayer oxide scales with an outer iron oxides layer grew rapidly in the first-stage exposure, and acted as a barrier for the diffusing species in the second-stage exposure. Herein, the scaling rate in the second-stage exposure (oxygen) would be controlled by the diffusion process. The thickening of the outer hematite layer and the much lower scaling rate may indicate that the inward transport of oxygen is important [11] and its diffusing ability is much slower than hydroxyl species in steam.

## 4. Experimental

Commercial ferritic/martensitic P92 was used in the present work and its chemical composition is Fe-0.1C-0.5Si-0.5Mn-0.4Ni-9.5Cr-0.6Mo-0.1Nb-0.3Cu-2W (in weight percent). P92 tube as received was cut into coupons with dimensions of 10 × 15 × 2 mm^3^. All the coupons were ground to 1000# with SiC papers, and cleaned ultra-sonically by distilled water and ethanol for 15 min in subsequence.

Isothermal oxidation experiments in the range of 600–800 °C were conducted in a TGA system (Thermo Fisher Scientific, Karlsruhe, Germany) where steam or oxygen could be introduced [14]. Flowing steam was generated by pumping ultra-purified water (resistivity is greater than 10 MΩ·cm) containing 7–9 ppm (wt%) dissolved oxygen into pre-heater at the flow rate 1 mL/min. The purity of oxygen is greater than 99.995%. Two-stage oxidation tests denoted as “O_2_ then H_2_O” and “H_2_O then O_2_” were also performed in the TGA system where oxidation temperature was maintained at 700 °C. Between the two stages, high purity argon was flowed to purge the oxidizing gas in the first stage. For each stage, 5 h exposure was used. In all cases, the heating and cooling rate are 80 °C/min and −5 °C/min respectively.

The oxidized specimens were characterized carefully in terms of scale microstructure and phase composition. Scale microstructures were investigated by OM (Zeiss, Jena, Germany) and SEM (FEI, Hillsboro, TX, USA). Oxide phases were identified by XRD (PANalytical, Almelo, The Netherlands). The outmost surface oxides formed in steam and the thin oxide scales formed in oxygen were analyzed by Grazing Incidence XRD (GIXRD) with an incidence angle of 0.5°.

## 5. Conclusions

Based on the experimental results, it can be concluded that:

(1) Within the studied temperature range, the scaling rates of P92 in pure steam were significantly increased compared to those in pure oxygen. Accordingly, non-protective oxide scales composed of an outer iron oxide layer (including hematite and magnetite), a middle Fe–Cr oxides layer and internal Cr-rich oxides formed in pure steam rather than more protective scales in pure oxygen. It is considered that the enhanced oxidation in pure steam was attributed to the higher affinity of iron to steam which resulted in the increase of critical chromium concentration in Fe–Cr alloys required to form protective chromia scale;
(2) Two-stage exposure at 700 °C showed a significant influence of gas switch on the scaling kinetics and scale microstructure of P92. It can be speculated that the breakaway of the initial protective oxide scale would be resulted from the hydrogen effect. Although the inward transport of anions through multilayer oxide scales was important in both environments, the migration of hydroxyl seemed much easier.

## Figures and Tables

**Table 1.** Summary of parabolic rate constants.

*T*/°C	k_p_ (steam)/(mg^2^·cm^−4^·h)	k_p_ (oxygen)/(mg^2^·cm^−4^·h)
600	0.11	–
700	1.70	3.67 × 10^−4^
800	4.46	5.40 × 10^−3^, 7.88 × 10^−2^
